# Personal

**Published:** 1907-11

**Authors:** 


### PERSONAL.

---

Dr. Frederic C. Curtis, of Albany, President of the Medical Society of the State of New York, paid a visit to Buffalo and attended the sessions of the Sanitary Conference held October 16-18, 1907.

---

Dr. Carlton C. Frederick, of Buffalo, was elected president of the Medical Association of Central New York at its fortieth annual meeting held at Rochester, October 15, 1907.

Dr. James A. Gibson, of Buffalo, who has been spending the summer in Europe, accompanied by his wife and daughter, has returned to his home and resumed his professional practice.

---

Dr. C. G. Leo-Wolf, of Niagara Falls, N. Y., has removed his offices to rooms 300, 301 and 302 in the Elderfield-Hartshorn building, 44 Falls Street. Hours: 9 to 10; 2 to 4 and 7.30 to 8.30. Telephones: Bell, 244; Home, 254. His residence is at 105 Jefferson Avenue.

---

Mr. William Whitford, medical stenographic reporter, of Chicago, was elected president of the National Shorthand Reporters' Association at the ninth annual meeting, held at Biltmore, near Asheville, N. C., August 6-9, 1907. He read a paper at the meeting, embodying twenty years of observation and researches on the rate of public speaking.

---

Dr. William Gaertner, of Buffalo, was an official delegate from the local body to the National German-American Alliance, which held its convention in New York, October 4-7, 1907. About 400 delegates were in attendance. A dinner was given at the Liederkranz Club, Saturday evening, October 5.

---

Dr. Esther Pohl has been elected city health officer of Portland, Ore. She will receive a salary of $3,000 a year. She was the first woman to enter the Oregon Medical College, and since being graduated has taken post-graduate courses in Baltimore and New York. She has also taken a degree in the Vienna University.

---

Dr. Edward Clark, of Buffalo, was elected president of the Medical Society of the County of Erie at its annual meeting held October 14, 1907. Dr. Clark has been active in the work of the society for more than twenty years and during much of the time has been treasurer. His elevation to the presidency should be satisfactory to every member of the society.

---

Dr. Joseph W. Grosvenor, of Buffalo, read a paper entitled, The soldier as a total abstainer from alcoholic beverages, before the American Academy of Medicine, at Atlantic City, June 1, 1907.

---

Dr. Jane W. Carroll, of Buffalo, read a paper before the medical section of the National Fraternal Congress, which held its annual meeting in this city August 19, 1907. The title of Dr. Carroll's paper was, Are cases of appendectomy in women suitable risks for fraternal insurance?

Dr. Robert Spann Cathcart, of Charleston, S. C., announces to the medical profession that after the first of October, nineteen hundred and seven he will confine his practice exclusively to gynecology and general surgery. Consultation by appointment. Telephone, 278. Fifty-five Hasell Street.

Dr. Charles Lybrand Bonifield, of Cincinnati, was elected president of the Ohio State Medical Association at its recent annual meeting.

